# Correction to “Dwelling in prolonged grief: Resting state functional connectivity during oxytocin and placebo administration”

**DOI:** 10.1002/hbm.26594

**Published:** 2024-02-09

Seeley, S. H., Andrews‐Hanna, J. R., Allen, J. J. B., & O'Connor, M.‐F. (2023). Dwelling in prolonged grief: Resting state functional connectivity during oxytocin and placebo administration. *Human Brain Mapping*, *44*(1), 245–257. https://doi.org/10.1002/hbm.26071

In the first paragraph of the Discussion (4.1 Summary and implications), our summary of results stated “higher resting state functional connectivity in the placebo session was associated with *fewer* prolonged grief symptoms.” This should have read “higher resting state functional connectivity in the placebo session was associated with *higher* prolonged grief symptoms.”

Results and conclusions stated elsewhere in the manuscript are unaffected by this correction and remain as described. We apologize for the error.

